# Clinical Significance and Outcome in Patients with Asymptomatic Versus Symptomatic Subsegmental Pulmonary Embolism

**DOI:** 10.3390/jcm12041640

**Published:** 2023-02-18

**Authors:** Ana Rodríguez-Cobo, Carmen Fernández-Capitán, Yale Tung-Chen, Giorgina Salgueiro-Origlia, Aitor Ballaz, Cristiano Bortoluzzi, Gabrielle Sarlon-Bartoli, Maria Lourdes Pesce, Dally Najib, Manuel Monreal, The RIETE Investigators

**Affiliations:** 1 Department of Internal Medicine, Hospital de Madrid Norte Sanchinarro, C. de Oña, 10, 28050 Madrid, Spain; 2 Department of Internal Medicine, Hospital Universitario La Paz, Paseo Castellana 241, 28046 Madrid, Spain; 3 Department of Pneumonology, Hospital de Galdakao, Labeaga Auzoa, 48960 Galdakao, Spain; 4 Department of Internal Medicine, Ospedale SS. Giovanni e Paolo di Venezia, Sestiere Castello, 6777, 30122 Venice, Italy; 5 Department of Vascular Medicine and Arterial Hypertension, Hôpital de la Timone, 264 Rue Saint-Pierre, 13005 Marseille, France; 6 Department of Internal Medicine, Hospital General Universitario de Elda, Ctra. Sax-La Torreta, S/N, 03600 Elda, Spain; 7 Department of Haematology, Hematology Institute of ZIV Medical Center, Derech HaRambam, Safed 64239, Israel; 8 Chair for the Study of Thromboembolic Disease, Faculty of Health Sciences, UCAM-Universidad Católica San Antonio de Murcia, CIBER Enfermedades Respiratorias (CIBERES), 30627 Madrid, Spain

**Keywords:** pulmonary embolism, venous thrombosis, anticoagulants, bleeding

## Abstract

The clinical significance and optimal therapy of patients with subsegmental pulmonary embolism (SSPE) remain controversial. We used the data in the RIETE Registry to compare the baseline characteristics, treatment, and outcomes during anticoagulation and after its discontinuation in patients with asymptomatic vs. symptomatic SSPE. From January 2009 to September 2022, there were 2135 patients with a first episode of SSPE, of whom 160 (7.5%) were asymptomatic. Most patients in both subgroups received anticoagulant therapy (97% vs. 99.4%, respectively). During anticoagulation, 14 patients developed symptomatic pulmonary embolism (PE) recurrences, 28 lower-limb deep vein thrombosis (DVT), 54 bled, and 242 died. The patients with asymptomatic SSPE had similar rates of symptomatic PE recurrences (hazard ratio (HR): 2.46; 95% CI: 0.37–9.74), DVT (HR: 0.53; 95% CI: 0.03–2.80), or major bleeding (HR: 0.85; 95% CI: 0.21–2.42) to those with symptomatic SSPE, but had a higher mortality rate (HR: 1.59; 95% CI: 1.25–2.94). The rate of major bleeding outweighed the rate of PE recurrences (54 major bleeds vs. 14 PE recurrences), and the rate of fatal bleeds outweighed the rate of fatal PE recurrences (12 vs. 6 deaths). After discontinuing anticoagulation, the patients with asymptomatic SSPE had a similar rate of PE recurrences (HR: 1.27; 95% CI: 0.20–4.55) and a non-significantly higher mortality rate (HR: 2.06; 95% CI: 0.92–4.10). The patients with asymptomatic SSPE had similar rates of PE recurrences to those with symptomatic SSPE, during and after discontinuing anticoagulation. The unexpectedly higher rate of major bleeding than recurrences highlights the need for randomized trials to find the best management.

## 1. Introduction

In recent decades, several studies have been conducted that challenged the diagnosis and treatment of patients with acute symptomatic pulmonary embolism (PE) [1,2,3,4]. However, a few questions remain unsolved, including the clinical significance and optimal therapy of patients with asymptomatic PE or those with subsegmental PE (SSPE). Prior studies did not specifically focus on these aspects, most likely because they were underpowered to evaluate the influence of asymptomatic PE on the outcome, or because of some uncertainty about the correct diagnosis of SSPE [5,6]. As a consequence, the current guidelines provide no specific recommendations regarding anticoagulant therapy for patients with asymptomatic PE or SSPE or suggest that individual patient risk profiles and preferences should guide the decision [5,6,7].

The RIETE (Registro Informatizado de Enfermedad TromboEmbólica) registry is an ongoing, international, observational registry of consecutive patients with objectively confirmed acute venous thromboembolism (VTE). The data from this registry have been used to evaluate outcomes after acute VTE, such as the frequency of VTE recurrences, bleeding and mortality, and risk factors for these outcomes [8,9]. In the current study, we aimed to compare the clinical characteristics, treatment, and outcomes of patients with incidentally found asymptomatic SSPE vs. those with symptomatic SSPE.

## 2. Materials and Methods

### 2.1. Data Source

We used data from the RIETE registry, which prospectively collects information on consecutive patients with objectively confirmed VTE (ClinicalTrials.gov identifier, NCT02832245). The design and conduct of the RIETE registry have been described previously [10]. All the patients provided written or oral informed consent for participation in the registry per the local ethics committee requirements. The quality of the data collection was regularly monitored and documented via periodic visits to participating hospitals and electronically. The local coordinators resolved any inconsistency or error.

### 2.2. Inclusion Criteria

The information on the angiographic localization of the pulmonary emboli on contrast-enhanced CT-scans was included in RIETE in January 2009. Thus, the consecutive patients with SSPE confirmed by helical CT-scan from January 2009 to September 2022 were considered for this analysis. The patients were excluded if they were currently participating in a blind therapeutic clinical trial.

### 2.3. Study Design

The major outcome was the occurrence of symptomatic, objectively confirmed pulmonary embolism (PE) recurrences appearing during anticoagulation or after its discontinuation in patients with asymptomatic SSPE vs. those with symptomatic SSPE. We considered asymptomatic patients with the absence of VTE signs and symptoms attributable to PE or DVT. All episodes of clinically suspected PE recurrences were investigated by repeat helical CT. The secondary outcomes were symptomatic, objectively confirmed deep vein thrombosis (DVT) in the lower limbs, major bleeding, and all-cause death. All episodes of clinically suspected DVT were investigated using compression ultrasonography (CUS). The bleeding complications were classed as major if they were overt and required a transfusion of two units of blood or more; were retroperitoneal, spinal, or intracranial; or they were fatal. Fatal PE, in the absence of autopsy, was defined as any death appearing within ten days after symptomatic PE diagnosis in the absence of any alternative cause of death. Fatal bleeding was considered any death occurring within ten days after a major bleed, in the absence of any alternative cause of death.

### 2.4. Treatment and Follow-Up

The patients were managed according to the clinical practice of each participating center. The drug, dose, and duration of anticoagulant therapy were recorded. The decision on the type and duration of therapy was left to the attending physicians. As RIETE is not an interventional study, the duration of anticoagulation may be variable based on the patients’ clinical status and decisions by the treating clinicians. The patients were followed-up in the outpatient clinic (or through telephone interviews with patients who could not attend a clinic visit). During each visit, any signs or symptoms suggesting VTE recurrences were noted. All the outcomes were classified as reported by the clinical centers.

### 2.5. Study Variables

The following variables were recorded in RIETE: patient’s characteristics; VTE signs and symptoms at baseline; clinical status, including any coexisting or underlying conditions such as chronic heart or lung disease, recent (<30 days before) major bleeding, anemia, or renal insufficiency; concomitant disorders; risk factors for VTE, including the use of estrogens; concomitant drugs; the treatment received upon SSPE diagnosis (drugs, doses, and duration); and the outcomes during and after discontinuing anticoagulant therapy. The immobilized patients were defined as non-surgical patients that had been immobilized (i.e., total bed rest with or without bathroom privileges) for ≥4 days in the 2 months before the VTE diagnosis. The surgical patients were defined as those that had undergone an operation in the 2 months before the index VTE. Active cancer was defined as newly diagnosed cancer (<3 months before) or when receiving anti-neoplastic treatment of any type (i.e., chemotherapy, radiotherapy, surgery, hormonal, support therapy, or combined therapies). Anemia was defined as hemoglobin levels < 12 g/dL. The creatinine clearance (CrCl) levels at baseline were calculated using the Cockcroft and Gault formula. The RIETE registry restricted all values of these variables to the nearest recorded to the time of VTE diagnosis.

### 2.6. Statistical Analysis

The categorical variables were reported as frequency counts (percentages) and compared using the chi-square test (two-sided) and Fisher’s Exact Test (two-sided). The continuous variables were reported as mean and standard deviation (or median with interquartile range, if not normally distributed), and compared using the Student’s *t*-test. The incidence rates of all the VTE-related outcomes (PE recurrences, DVT, major bleeding, or death) were calculated as a number of events per 100 patient years. The incidence rate of symptomatic PE recurrences and all-cause death were compared between patients presenting with asymptomatic SSPE versus symptomatic SSPE using Cox proportional hazard regression models. The hazard ratios (HR) with the corresponding 95% confidence intervals (CI) were used as the effect measure. We used competing-risk analysis for death to compare the risk of developing symptomatic PE recurrences between subgroups. The covariates included in the adjusted models were those considered clinically significant or those for which a significant difference (a threshold *p*-value of <0.1) was found. The variables entered in the models included sex, age, body weight, risk factors for SSPE (i.e., active cancer, transient risk factors, or unprovoked), diabetes, hypertension, chronic heart failure, chronic lung disease, prior myocardial infarction, ischemic stroke or peripheral artery disease, recent (<30 days before) major bleeding, and discontinuation of anticoagulant therapy.

All the analyses were conducted using the statistical IBM SPSS software v25.0 (SPSS Inc., Chicago, IL, USA).

## 3. Results

From January 2009 to October 2022, 2135 patients were diagnosed with the first episode of SSPE in RIETE. Of these, 160 (7.5%) were asymptomatic. The Patients with asymptomatic SSPE were older to those with symptomatic SSPE (68 ± 15 vs. 64 ± 17 years, respectively), more likely to have active cancer (43% vs. 16%) or recent surgery (16% vs. 11%), and less likely to have unprovoked VTE (31% vs. 49%), as shown in Table 1.

There were no differences in the proportion of patients initially presenting with hypotension or tachycardia, nor in those undergoing CUS in the lower limbs (39% vs. 45%) (Table 2). Most patients in both subgroups (83% each) received initial therapy with low-molecular-weight heparin (LMWH). Only two patients with symptomatic SSPE (0.1%) received thrombolytic drugs (Table 2). For long-term therapy, 52% of the patients with asymptomatic SSPE received LMWH, compared to 28% of those with symptomatic SSPE.

**Figure 1:**
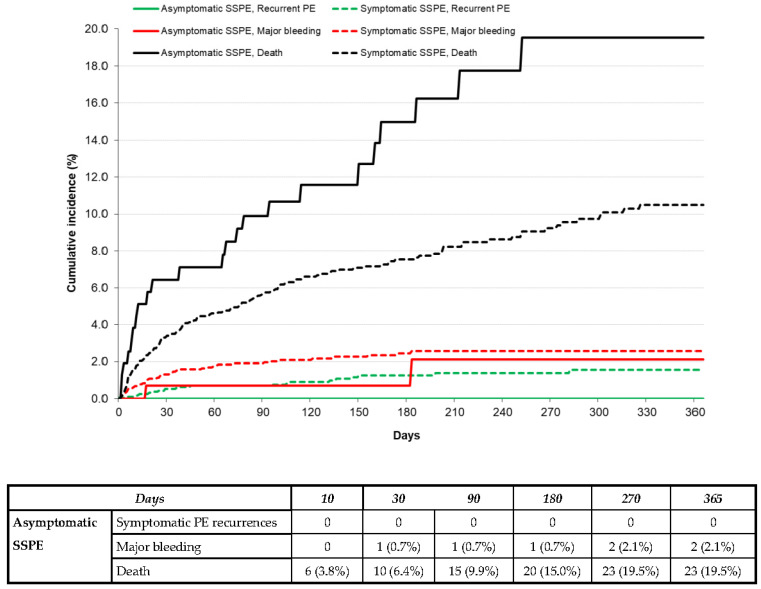
Cumulative incidence rate of symptomatic PE recurrences, major bleeding, or death during the course of anticoagulation in patients with asymptomatic vs. symptomatic SSPE.

During anticoagulation, 14 patients developed symptomatic PE recurrences, 28 had lower-limb DVT, 54 had major bleeding (gastrointestinal 18 and intracranial 10), and 242 died (fatal PE recurrences 6 and fatal bleeding 12). The patients with asymptomatic SSPE had similar rates of symptomatic PE recurrences (hazard ratio (HR): 2.46; 95% CI: 0.37–9.74), DVT (HR: 0.53; 95% CI: 0.03–2.80) or major bleeding (HR: 0.85; 95% CI: 0.21–2.42) to those with symptomatic SSPE but had a higher mortality rate (HR: 1.59; 95% CI: 1.25–2.94), as shown in Table 3 and Figure 1.

**Figure 2:**
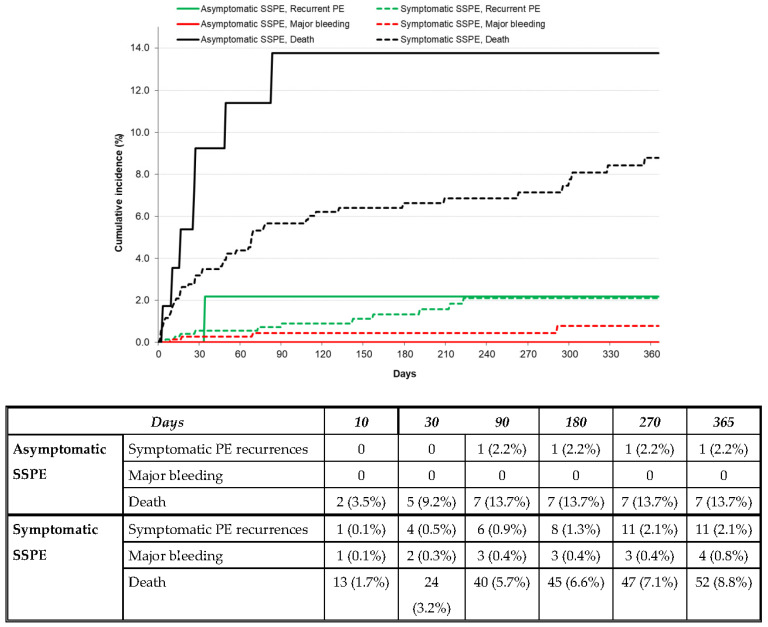
Cumulative incidence rate of symptomatic PE recurrences, major bleeding, or death after discontinuing anticoagulation in patients with asymptomatic vs. symptomatic SSPE.

After discontinuing anticoagulant therapy, 28 patients developed symptomatic PE recurrences, 13 had lower-limb DVT, 6 had major bleeding, and 75 died (fatal PE 1 and fatal bleeding 5). The patients with asymptomatic SSPE had a similar rate of PE recurrences (HR: 1.27; 95% CI: 0.20–4.55) and a non-significantly higher mortality rate (HR: 2.06; 95% CI: 0.92–4.10) to those with symptomatic SSPE (Table 3 and Figure 2). No patient with asymptomatic SSPE developed DVT or major bleeding after discontinuing anticoagulation (Table 3).

Over half of the patients (28 of 51, 55%) that suffered major bleeding were receiving LMWH at the moment of bleeding (Table 4).

None of the 62 patients with asymptomatic SSPE who underwent lower-limb CUS developed symptomatic PE recurrences during anticoagulant therapy, nor after its discontinuation (Table 5).

In the multivariable analysis, we found no significant differences in the risk of developing either symptomatic PE recurrences (adjusted HR: 0.70; 95% CI: 0.22–2.24) or death (adjusted HR: 1.07; 95% CI: 0.73–1.56) between the patients initially presenting with asymptomatic vs. symptomatic SSPE (Table 6).

## 4. Discussion

Our findings, obtained from a large cohort of patients with SSPE, reveal that the incidence rate of symptomatic PE recurrences was similar in patients initially presenting with asymptomatic or symptomatic PE, both during anticoagulation (1.98 vs. 0.81 per 100 patient years) or after its discontinuation (4.38 vs. 3.45 per 100 patient years). To elaborate, 1 in every 6 patients presenting with PE recurrences (7 of 43, 16%) died of the PE recurrence. Even higher if considering only the treatment period (6 of 14, 43%). Thus, its clinical significance should not be underestimated. Interestingly, however, the rate of major bleeding during anticoagulation outweighed the rate of symptomatic PE recurrences (54 major bleeds vs. 14 PE recurrences), and the rate of fatal bleeds outweighed the rate of fatal PE recurrences (12 vs. 6 deaths, respectively). Thus, we should be cautious before prescribing full-dose anticoagulants to this patient population. After discontinuing anticoagulant therapy, there were more PE recurrences than major bleeds (29 vs. 6, respectively). However, the rate of fatal bleeds still exceeded the rate of fatal PE recurrences (five vs. one death, respectively).

A similar rate of PE recurrences during anticoagulation in the patients with asymptomatic vs. symptomatic SSPE was also reported in a study performed at the Mayo Thrombophilia Clinic (2.18 vs. 2.00 per 100 person years, respectively) [11]. As in our cohort, the patients with asymptomatic SSPE were more likely to have cancer than those with symptomatic SSPE. The higher rate of major bleeding in our cohort was also found in a recent meta-analysis of 14 studies on patients with SSPE [12], thus highlighting the need for randomized trials to determine the benefit or harm of anticoagulation in this patient population.

The current guidelines from the American College of Chest Physicians (ACCP) recommend bilateral lower-limb ultrasonography in patients with SSPE to exclude proximal DVT [13]. In those with proximal DVT, anticoagulant therapy is recommended; those without DVT suggest clinical surveillance if there is a low risk for recurrent VTE and depending on coexisting bleeding risk. We failed to find any PE recurrence in the patients with SSPE and proximal DVT. In a recent study on 292 SSPE patients without proximal DVT not receiving anticoagulants, the rate of PE recurrences was 3.1% and major bleeding 0.7% at 3 months [14], which would suggest some benefit for anticoagulation. However, the study only included low-risk VTE patients and was stopped earlier than planned [14]. The European Society of Cardiology (ESC) guidelines suggest the anticoagulation of incidental PE in oncologic patients, and they emphasize the lack of evidence in other patient subgroups [15]. Finally, a recent Cochrane systematic review concluded that there is no evidence to support any recommendation on the use of anticoagulation in these patients [16].

Several limitations of our study need to be acknowledged. First, it is not possible to determine why physicians decided not to treat some patients with SSPE. The reasons could range from the fact that the patients with SSPE were healthier and with a lower risk for VTE, or had end-stage medical conditions or a high bleeding risk. Second, the high risk of misclassification was due to a low interrater agreement for diagnosing SSPE among radiologists [17,18,19]. Third, the vast majority of patients in our cohort received anticoagulant therapy, and the lack of a similar sample of SSPE patients without anticoagulation can cause it to be difficult to draw a meaningful conclusion on the impact of anticoagulation. Despite these limitations, our data have been recruited from different hospitals and countries, allowing precision in our results, and thus impacting the generalizability of our study. [10] Our results suggest that the patients with asymptomatic SSPE should receive a similar treatment strategy as those with symptomatic SSPE. However, until further controlled clinical trials provide better evidence of the best management, the treatment should be individualized and best left to clinical judgment or patient-shared decision-making.

## 5. Conclusions

We conclude that the patients with asymptomatic SSPE had similar rates of PE recurrences to those with symptomatic SSPE, during and after discontinuing anticoagulation. This suggests that they should receive similar treatment strategies. Yet, the unexpectedly high rate of major bleeding highlights the need for controlled clinical trials to provide better evidence of the best management.

## Figures and Tables

**Table 1:** Baseline characteristics of patients with symptomatic vs. asymptomatic SSPE.

	Asymptomatic SSPE	Symptomatic SSPE	Odds Ratio (95% CI)
Patients, N	160	1975	
Demographics,			
Male gender	83 (52%)	987 (50%)	1.08 (0.78–1.49)
Age (mean years ± SD)	68 ± 15 ^†^	64 ± 17	*p* = 0.009
Body weight (mean kg ± SD)	72 ± 15 *	76 ± 17	*p* = 0.039
Comorbidities,			
Hypertension	65 (41%)	871 (44%)	0.87 (0.62–1.20)
Diabetes	20 (13%)	294 (16%)	0.82 (0.50–1.34)
Atrial fibrillation	7 (4.4%)	105 (5.3%)	0.81 (0.37–1.78)
Chronic lung disease	18 (11%)	292 (15%)	0.73 (0.44–1.21)
Chronic heart failure	8 (5.0%)	182 (9.2%)	0.52 (0.25–1.07)
Prior myocardial infarction	6 (3.8%)	140 (7.1%)	0.51 (0.22–1.18)
Prior ischemic stroke	15 (9.4%)	117 (5.9%)	1.64 (0.94–2.89)
Peripheral artery disease	6 (4.0%)	67 (3.6%)	1.12 (0.48–2.62)
Recent major bleeding	8 (5.0%)	60 (3.0%)	1.68 (0.79–3.58)
Recent COVID-19 infection	14 (8.8%)	278 (14.0%)	0.59 (0.33–1.03)
Risk factors for PE,			
Active cancer	68 (43%) ^‡^	310 (16%)	3.97 (2.84–5.56)
Recent immobilization	35 (22%)	478 (24%)	0.88 (0.59–1.29)
Recent surgery	26 (16%)	215 (11%)	1.59 (1.02–2.47)
Hormonal use	5 (3.2%)	111 (5.8%)	0.53 (0.21–1.32)
Recent travel	2 (1.3%)	49 (2.6%)	0.49 (0.12–2.04)
Pregnancy	1 (0.63%)	23 (1.2%)	0.53 (0.07–3.98)
None of the above (unprovoked)	49 (31%) ^‡^	962 (49%)	0.46 (0.33–0.66)

Differences between patients with asymptomatic vs. symptomatic SSPE: * *p* < 0.05; ^†^ *p* < 0.01; ^‡^ *p* < 0.001. Abbreviations: SSPE, subsegmental pulmonary embolism; SD, standard deviation; PE, pulmonary embolism; and CI, confidence intervals.

**Table 2:** Signs, symptoms, diagnostic tests, and therapeutic strategies.

	Asymptomatic SSPE	Symptomatic SSPE	Odds Ratio (95% CI)
Patients, N	160	1975	
Signs/symptoms,			
Dyspnea	0 ^‡^	1337 (68%)	-
Chest pain	0 ^‡^	796 (40%)	-
Syncope	0 ^‡^	165 (8.4%)	-
Hemoptysis	0 ^‡^	81 (4.1%)	-
Lower-limb pain	0 ^‡^	409 (21%)	-
Lower-limb swelling	0 ^‡^	387 (20%)	-
SBP levels < 100 mmHg	10 (7.0%)	99 (5.0%)	1.43 (0.73–2.80)
Heart rate > 110 bpm	16 (11%)	214 (11%)	1.00 (0.58–1.71)
Blood levels,			
Sat O_2_ levels < 90% (n = 876)	7 (13%)	156 (19%)	0.64 (0.28–1.43)
Positive D-dimer (n = 1491)	68 (96%)	1339 (94%)	1.37 (0.42–4.45)
Raised troponin (n = 965)	15 (32%)	200 (22%)	1.68 (0.89–3.17)
Lower-limb ultrasound,			
Proximal DVT	14 (8.8%)	278 (14%)	0.59 (0.33–1.03)
Distal DVT	11 (6.9%)	111 (5.6%)	1.24 (0.65–2.35)
Normal	37 (23%)	502 (25%)	0.88 (0.60–1.29)
Not performed	98 (61%) *	1042 (53%)	1.42 (1.02–1.97)
Prognostic scoring,			
Simplified PESI < 1 points	49 (31%) ^‡^	909 (46%)	0.52 (0.37–0.73)
Initial therapy,			
Low-molecular-weight heparin	133 (83%)	1640 (83%)	1.01 (0.65–1.55)
Unfractionated heparin	10 (6.3%)	82 (4.2%)	1.54 (0.78–3.03)
Fondaparinux	3 (1.9%)	27 (1.4%)	1.38 (0.41–4.59)
Direct oral anticoagulants	5 (3.3%) ^†^	187 (9.5%)	0.32 (0.13–0.80)
Rivaroxaban	2 (1.3%) *	113 (5.7%)	0.21 (0.05–0.85)
Apixaban	2 (1.3%)	59 (3%)	0.41 (0.10–1.70)
Thrombolytics	0	2 (0.10%)	-
No initial therapy	5 (3.1%)	12 (0.6%)	5.17 (1.84–15.2)
Long-term therapy,			
Low-molecular-weight heparin	83 (52%) ^‡^	549 (28%)	2.80 (2.02–3.88)
Vitamin K antagonists	33 (21%) ^‡^	753 (38%)	0.42 (0.28–0.63)
Fondaparinux	0	12 (0.61%)	-
Direct oral anticoagulants	31 (21%) *	585 (31%)	0.60 (0.40–0.91)
Rivaroxaban	12 (7.5%) ^†^	299 (15%)	0.45 (0.25–0.83)
Apixaban	13 (8.1%)	195 (9.9%)	0.81 (0.45–1.45)
Edoxaban	5 (3.1%)	68 (3.4%)	0.90 (0.36–2.28)
Dabigatran	1 (0.6%)	23 (1.2%)	0.53 (0.07–3.98)
No long-term therapy	4 (2.5%)	18 (0.9%)	2.78 (0.93–8.34)

Differences between patients with asymptomatic vs. symptomatic SSPE: * *p* < 0.05; ^†^ *p* < 0.01; ^‡^ *p* < 0.001. Abbreviations: SSPE, subsegmental pulmonary embolism; SBP, systolic blood pressure; bpm, beats per minute; DVT, deep vein thrombosis; PESI, pulmonary embolism severity index; and CI, confidence intervals.

**Table 3:** Clinical outcomes during the course of anticoagulant therapy and after its discontinuation.

	Asymptomatic SSPE		Symptomatic SSPE		Hazard Ratio (95% CI)
	N	Events per 100 Patient Years	N	Events per 100 Patient Years	
During anticoagulation					
Patients, N	160		1975		
Duration of therapy,					
Median days (IQR)	148 (94–306)		173 (101–306)		
Mean days ± SD	240 ± 267		277 ± 382		*p* = 0.101
Outcomes,					
Recurrent VTE	3	2.98 (0.76–8.10)	38	2.60 (1.86–3.53)	1.15 (0.28–3.32)
Recurrent PE	2	1.98 (0.33–6.55)	12	0.81 (0.44–1.37)	2.46 (0.37–9.74)
Recurrent DVT	1	0.97 (0.05–4.79)	27	1.84 (1.24–2.64)	0.53 (0.03–2.80)
Major bleeding	3	2.91 (0.74–7.93)	51	3.44 (2.59–4.48)	0.85 (0.21–2.42)
Gastrointestinal	2	1.94 (0.33–6.42)	16	1.07 (0.64–1.71)	1.81 (0.28–6.85)
Intracranial	1	0.97 (0.05–4.79)	9	0.60 (0.29–1.10)	1.61 (0.07–9.82)
Death	24	23.3 (15.3–34.1) ^†^	179	12.0 (10.3–13.8)	1.95 (1.25–2.94)
Fatal PE	0	-	6	0.40 (0.16–0.83)	-
Fatal bleeding	1	0.97 (0.05–4.79)	11	0.74 (0.39–1.28)	1.32 (0.06–7.73)
After discontinuing anticoagulant therapy					
Patients, N	58		781		
Duration of therapy,					
Median days (IQR)	154 (43–331)		198 (75–475)		
Mean days ± SD	287 ± 419		367 ± 485		*p* = 0.169
Outcomes,					
Recurrent VTE	2	4.38 (0.73–14.5)	40	5.16 (3.74–6.96)	0.85 (0.14–2.96)
Recurrent PE	2	4.38 (0.73–14.48)	27	3.45 (2.32–4.95)	1.27 (0.20–4.55)
Recurrent DVT	0	-	13	1.67 (0.93–2.79)	-
Major bleeding	0	-	6	0.77 (0.31–1.59)	-
Gastrointestinal	0	-	4	0.51 (0.16–1.23)	-
Intracranial	0	-	0	-	-
Death	8	17.5 (8.14–33.3)	67	8.53 (6.66–10.8)	2.06 (0.92–4.10)
Causes of death					
Fatal PE	0	-	1	0.13 (0.01–0.63)	-
Fatal bleeding	0	-	5	0.64 (0.23–1.41)	-

Differences between patients with asymptomatic vs. symptomatic SSPE. ^†^ *p* < 0.01. Abbreviations: SSPE, subsegmental pulmonary embolism; IQR, inter-quartile range; SD, standard deviation; PE, pulmonary embolism; DVT, deep vein thrombosis; and CI, confidence intervals.

**Table 4:** Recurrent venous thromboembolism and major bleeding during the course of anticoagulant therapy, according to the drugs used.

	Asymptomatic SSPE		Symptomatic SSPE		Hazard Ratio (95% CI)
	N	Events per 100 Patient Years	N	Events per 100 Patient Years	
In patients on LMWH, N					
Symptomatic PE	0	-	3	0.52 (0.13–1.41)	-
Symptomatic DVT	0	-	2	0.34 (0.06–1.14)	-
Major bleeding	3	4.80 (1.22–13.1)	28	4.85 (3.29–6.92)	0.99 (0.24–2.93)
*In patients on VKAs, N*					
Symptomatic PE	1	3.18 (0.16–15.7)	24	3.16 (2.07–4.63)	1.01 (0.05–5.37)
Symptomatic DVT	2	6.83 (1.15–22.6)	10	1.29 (0.65–2.29)	5.31 (0.79–21.8)
Major bleeding	0	-	18	2.32 (1.42–3.59)	-
In patients on DOACs, N					
Symptomatic PE	0	-	0	-	-
Symptomatic DVT	0	-	0	-	-
Major bleeding	0	-	3	2.43 (0.62–6.62)	-
In patients on other drugs, N					
Symptomatic PE	0	-	0	-	-
Symptomatic DVT	0	-	0	-	-
Major bleeding	0	-	2	33.0 (5.54–109.2)	-

Abbreviations: SSPE, subsegmental pulmonary embolism; LMWH, low-molecular-weight heparin; VKAs, vitamin K antagonists; DOACs, direct oral anticoagulants; PE, pulmonary embolism; DVT, deep vein thrombosis; and CI, confidence intervals.

**Table 5:** Clinical outcomes during the course of anticoagulant therapy and after its discontinuation, according to the existence of concomitant lower-limb deep vein thrombosis. Patients with no compression ultrasound are not included.

	Asymptomatic SSPE				Symptomatic SSPE			
	Confirmed DVT		No DVT		Confirmed DVT		No DVT	
	N	Events per 100 Patient Years	N	Events per 100 Patient Years	N	Events per 100 Patient Years	N	Events per 100 Patient Years
During anticoagulation								
*Patients, N*	*25*		*37*		*389*		*502*	
Median days (IQR)	218 ± 253		240 ± 179		357 ± 586		275 ± 313	
Mean days ± SD	97 (38–257)		199 (98–342)		185 (104–349)		184 (102–345)	
Outcomes,								
Recurrent VTE	0	-	0	-	9	2.46 (1.20–4.51)	9	2.41 (1.18–4.43)
Recurrent PE	0	-	0	-	6	1.64 (0.66–3.40)	8	2.14 (1.00–4.07)
Recurrent DVT	0	-	0	-	4	1.05 (0.34–2.54)	1	0.26 (0.01–1.31)
Major bleeding	0	-	0	-	10	2.66 (1.35–4.75)	12	3.20 (1.73–5.44)
Gastrointestinal	0	-	0	-	4	1.06 (0.34–2.55)	5	1.33 (0.49–2.94)
Intracranial	0	-	0	-	2	0.53 (0.09–1.74)	1	0.26 (0.01–1.31)
Death	5	33.5 (12.3–74.3)	2	8.46 (1.42–27.9)	31	8.16 (5.64–11.4)	30	7.94 (5.46–11.2)
After discontinuing anticoagulant therapy								
*Patients, N*	*5*		*14*		*119*		*198*	
Median days (IQR)	184 ± 72		445 ± 627		452 ± 582		418 ± 533	
Mean days ± SD	199 (130–208)		162 (58–596)		206 (65–636)		243 (88–529)	
Outcomes,								
Recurrent VTE	0	-	0	-	10	7.21 (3.66–12.8)	10	4.42 (2.24–7.87)
Recurrent PE	0	-	0	-	4	2.74 (0.87–6.61)	9	3.97 (1.94–7.29)
Recurrent DVT	0	-	0	-	6	4.28 (1.74–8.91) *	1	0.44 (0.02–2.18) *
Major bleeding	0	-	0	-	0	-	1	0.44 (0.02–2.19)
Death	0	-	1	5.86 (0.29–28.9)	11	7.47 (3.93–13.0)	18	7.94 (4.85–12.3)

Differences between patients with asymptomatic vs. symptomatic SSPE: *p* < 0.05. Comparisons between patients with vs. without DVT on compression ultrasonography: * *p*-value < 0.05. Abbreviations: SSPE, subsegmental pulmonary embolism; IQR, inter-quartile range; SD, standard deviation; PE, pulmonary embolism; DVT, deep vein thrombosis; and CI, confidence intervals.

**Table 6:** Uni- and multivariable analyses for recurrent PE (using competing risk analysis for death) and for all-cause death during follow-up (either during anticoagulation or after its discontinuation).

	Symptomatic PE Recurrences		Death	
	Univariable	Multivariable	Univariable	Multivariable
Patients, N				
Demographics,				
Male gender	1.18 (0.70–1.98)	-	1.36 (1.06–1.76) *	0.67 (0.52–0.87) ^†^
Age >70 years	0.68 (0.39–1.17)	-	2.47 (1.90–3.19) ^‡^	1.81 (1.38–2.37) ^‡^
Body weight >75 kg	1.71 (1.01–2.89) *	1.77 (1.05–2.98) *	0.49 (0.38–0.65) ^‡^	0.59 (0.45–0.77) ^‡^
Comorbidities,				
Hypertension	1.35 (0.74–0.81)	-	1.33 (1.03–1.71) *	0.82 (0.62–1.07)
Diabetes	1.54 (0.81–2.93)	-	2.01 (1.47–2.74) ^‡^	1.63 (1.20–2.20) ^†^
Atrial fibrillation	1.20 (0.43–3.32)	-	2.62 (1.69–4.06) ^‡^	1.90 (1.26–2.87) ^†^
Chronic lung disease	1.32 (0.70–2.52)	-	2.16 (1.59–2.93) ^‡^	1.16 (0.86–1.57)
Chronic heart failure	0.61 (0.22–1.70)	-	2.20 (1.53–3.17) ^‡^	1.83 (1.26–2.66) ^†^
Prior myocardial infarction	0.41 (0.10–1.67)	-	2.17 (1.45–3.26) ^‡^	1.42 (0.96–2.11)
Prior ischemic stroke	1.50 (0.60–3.75)	-	2.50 (1.66–3.78) ^‡^	1.47 (0.99–2.18)
Peripheral artery disease	0.52 (0.07–3.81)	-	2.28 (1.32–3.94) ^†^	1.36 (0.81–2.28)
Recent major bleeding	1.32 (0.32–5.48)	-	1.61 (0.87–2.98)	-
Risk factors for SSPE,				
Unprovoked	Ref.	Ref.	Ref.	Ref.
Transient risk factors	0.70 (0.38–1.20)	-	1.90 (1.32–2.74) ^†^	2.15 (1.48–3.13) ^‡^
Active cancer	1.70 (0.95–3.03)	1.94 (1.08–3.50) *	12.2 (8.72–17.2) ^†^	10.9 (7.77–15.2) ^‡^
Initial SSPE presentation,				
Asymptomatic SSPE	0.77 (0.25–2.37)	0.70 (0.22–2.24)	1.83 (1.22–2.75) ^†^	1.07 (0.73–1.56)
Use of anticoagulant therapy,				
Off anticoagulation	1.69 (1.01–2.81) *	1.77 (1.07–2.91) *	0.54 (0.41–0.72) ^†^	0.50 (0.38–0.66) ^‡^

* *p* < 0.05; ^†^ *p* < 0.01; ^‡^ *p* < 0.001. Abbreviations: SSPE, subsegmental pulmonary embolism; Ref., reference.

## Data Availability

The authors confirm that the data supporting the findings of this study are available from the corresponding author, upon reasonable request.

## Appendix A

List of members of RIETE.

Coordinator of the RIETE Registry: Manuel Monreal.

RIETE Steering Committee Members: Paolo Prandoni, Benjamin Brenner, and Dominique Farge-Bancel.

RIETE National Coordinators: Raquel Barba (Spain), Pierpaolo Di Micco (Italy), Laurent Bertoletti (France), Sebastian Schellong (Germany), Inna Tzoran (Israel), Abilio Reis (Portugal), Marijan Bosevski (R. Macedonia), Henri Bounameaux (Switzerland), Radovan Malý (Czech Republic), Peter Verhamme (Belgium), Joseph A. Caprini (USA), and Hanh My Bui (Vietnam).

RIETE Registry Coordinating Center: S & H Medical Science Service.

Members of the RIETE Group

SPAIN: Adarraga MD, Alberich-Conesa A, Alonso-Carrillo J, Amado C, Amorós S, Arcelus JI, Ballaz A, Barba R, Barbagelata C, Barrón M, Barrón-Andrés B, Beddar-Chaib F, Blanco-Molina A, Chasco L, Criado J, del Toro J, De Ancos C, Demelo-Rodríguez P, De Juana-Izquierdo C, Díaz-Brasero AM, Díaz-Pedroche MC, Díaz-Peromingo JA, Dubois-Silva A, Escribano JC, Espósito F, Falgá C, Farfán-Sedano AI, Fernández-Capitán C, Fernández-Jiménez B, Fernández-Muixi J, Fernández-Reyes JL, Font C, Francisco I, Galeano-Valle F, García MA, García de Herreros M, García-Bragado F, García-González C, García-Ortega A, Gavín-Sebastián O, Gil-Díaz A, Gómez-Cuervo C, González-Martínez J, Grau E, Guirado L, Gutiérrez J, Hernández-Blasco L, Jaras MJ, Jiménez R, Jiménez D, Jou I, Joya MD, Lainez-Justo S, Latorre-Díez A, Lecumberri R, León-Ramírez JM, Lobo JL, López-De la Fuente M, López-Jiménez L, López-Miguel P, López-Núñez JJ, López-Reyez R, López-Ruiz A, López-Sáez JB, Lorente MA, Lorenzo A, Lumbierres M, Madridano O, Maestre A, Mas-Maresma L, Marcos M, Martín-Guerra JM, Martín-Martos F, Mellado M, Mena E, Mencía B, Mercado MI, Moisés J, Monreal M, Muñoz-Blanco A, Muñoz-Gamito G, Nieto JA, Núñez-Fernández MJ, Olid-Velilla M, Osorio J, Otalora S, Pacheco-Gómez N, Parra P, Pedrajas JM, Pérez-Ductor C, Pérez-Pérez JL, Peris ML, Pesce ML, Porras JA, Poyo-Molina J, Puchades R, Riera-Mestre A, Rivera-Civico F, Rivera-Gallego A, Roca M, Rosa V, Rodríguez-Cobo A, Rubio CM, Ruiz-Artacho P, Ruiz-Giménez N, Ruiz-Ruiz J, Salgueiro G, Sancho T, Sendín V, Sigüenza P, Soler S, Suárez-Rodríguez B, Suriñach JM, Tolosa C, Torres MI, Trujillo-Santos J, Uresandi F, Valle R, Varona JF, Vidal G, Villalobos A, Villares P, AUSTRIA: Ay C, Nopp S, Pabinger I, BELGIUM: Engelen M, Vanassche T, Verhamme P, BRAZIL: Yoo HHB, COLOMBIA: Arguello JD, Montenegro AC, Roa J, CZECH REPUBLIC: Hirmerova J, Malý R, FRANCE: Accassat S, Bertoletti L, Bura-Riviere A, Catella J, Chopard R, Couturaud F, Espitia O, Grange C, Leclercq B, Le Mao R, Mahé I, Moustafa F, Plaisance L, Poenou G, Sarlon-Bartoli G, Suchon P, Versini E, GERMANY: Schellong S, ISRAEL: Brenner B, Kenet G, Najib D, Tzoran I, IRAN: Sadeghipour P, ITALY: Basaglia M, Bilora F, Bortoluzzi C, Brandolin B, Ciammaichella M, Colaizzo D, Dentali F, Di Micco P, Giorgi-Pierfranceschi M, Grandone E, Imbalzano E, Merla S, Pesavento R, Prandoni P, Scarinzi P, Siniscalchi C, Taflaj B, Tufano A, Visonà A, Vo Hong N, Zalunardo B, LATVIA: Kigitovica D, Skride A, Zaicenko A, PORTUGAL: Carmo F, Fonseca S, Manuel M, Meireles J, Pinto S, REPUBLIC OF MACEDONIA: Bosevski M, Trajkova M, Zdraveska M, SWITZERLAND: Bounameaux H, Mazzolai L, UNITED KINGDOM: Aujayeb A, USA: Caprini JA, Weinberg I, VIETNAM: Bui HM.
